# The TGF-*β*1 Signaling Pathway as an Attractive Target in the Fibrosis Pathogenesis of Sjögren's Syndrome

**DOI:** 10.1155/2018/1965935

**Published:** 2018-11-26

**Authors:** Margherita Sisto, Loredana Lorusso, Giuseppe Ingravallo, Roberto Tamma, Domenico Ribatti, Sabrina Lisi

**Affiliations:** ^1^Department of Basic Medical Sciences, Neurosciences and Sensory Organs (SMBNOS), Section of Human Anatomy and Histology, University of Bari “Aldo Moro”, Bari, Italy; ^2^Department of Emergency and Organ Transplantation (DETO), Pathology Section, University of Bari “Aldo Moro”, Bari, Italy

## Abstract

Transforming growth factor *β*1 (TGF-*β*1) plays a crucial role *in the induction of fibrosis, often associated with chronic phases of inflammatory diseases* contributing to marked fibrotic changes that compromise normal organ function. The TGF-*β*1 signal exerts its biological effects via the TGF-*β*/SMAD/Snail signaling pathway, playing an important pathogenic role in several fibrotic diseases. It has as yet been poorly investigated in the chronic autoimmune disease Sjögren's syndrome (SS). Here, we firstly tested, by immunohistochemistry, whether the TGF-*β*1/SMAD/Snail signaling pathway is triggered in human pSS salivary glands (SGs). Next, healthy salivary gland epithelial cell (SGEC) cultures derived from healthy donors were exposed to TGF-*β*1 treatment, and the relative gene and protein levels of *SMAD2/3/4*, Snail, E-cadherin, vimentin, and collagen type I were compared by semiquantitative RT-PCR, quantitative real-time PCR, and Western blot analysis. We observed, both at gene and protein levels, higher expression of *SMAD2*, *3*, and *4* and Snail in the SGEC exposed by TGF-*β*1 compared to untreated healthy SGEC. Additionally, in TGF-*β*1-treated samples, we found a significant reduction in the epithelial phenotype marker E-cadherin and an increase in the mesenchymal phenotype markers vimentin and collagen type I compared to those in untreated SGEC, indicating that TGF-*β*1 induces the EMT via the TGF-*β*1/SMAD/Snail signaling pathway. Therefore, by using the specific TGF-*β* receptor 1 inhibitor SB-431542 in healthy SGEC treated with TGF-*β*1, we showed a significant reduction of the fibrosis markers vimentin and collagen type I while the epithelial marker E-cadherin returns to levels similar to untreated healthy SGEC. These data demonstrate that TGF-*β*1 is an important key factor in the transition phase from SG chronic inflammation to fibrotic disease. Characteristic changes in the morphology and function of TGF-*β*1-treated healthy SGEC further confirm that TGF-*β*1 plays a significant role in EMT-dependent fibrosis.

## 1. Introduction

Fibrosis is characterized by an excessive accumulation of extracellular matrix in the affected tissue, which often results in the destruction of the normal architecture and causes significant organ dysfunction. The fibrotic process occurs in various organs, and although it can have multiple causes, organ fibrosis typically results from chronic persistent inflammation induced by numerous different stimuli, such as chronic infections, ischemia, autoimmune diseases, and radiation exposure [1–4].

Therefore, close attention has been paid to the epithelial-mesenchymal transition (EMT) process and its involvement in the pathogenesis of fibrotic diseases [3, 5–7]. Injury triggered by the inflammatory wound healing cascade is tightly linked with fibrogenesis and was recently associated with atrophy and fibrosis of the salivary glands (SGs) [8–10]. Indeed, SG fibrosis typically occurs after repeated episodes of inflammation causing the constriction of secretory components and leading to hyposalivation and xerostomia [11], as in primary Sjögren's syndrome (pSS) [12]. pSS is a systemic rheumatic autoimmune disorder whose cardinal features are chronic, severe dry eyes and mouth and focal lymphocytic infiltrates in salivary and lacrimal gland tissue [13]. Fibrosis in the SGs of pSS patients has been observed [12], being a common consequence of tissue damage and inflammation and, in addition, an element of the pSS disease [12]. This mechanism is still not clear, but the prevailing hypothesis suggests that fibrogenic mediators produced by inflammatory and epithelial cells are implicated, in particular transforming growth factor- (TGF-) *β*1 [14]. TGF-*β*1 is the major driver of fibrosis in most forms of chronic inflammatory diseases. Inhibition of TGF-*β*1 isoform considerably attenuates fibrosis in a broad range of disease models, whereas an increased expression of TGF-*β*1 induces the fibrotic process [15]. Recent studies have demonstrated that TGF-*β*1 can induce renal fibrosis via activation of the SMAD signaling pathway, which results in the activation of myofibroblasts, excessive production of the extracellular matrix (ECM), and inhibition of ECM degradation [16]. The key role of SMAD proteins in the development of the fibrotic process is complex, featuring profibrotic and antifibrotic activities, and this mechanism is governed by an interplay between TGF-*β*1/SMADs and other signaling pathways [17].

Recent evidence from studies of other fibrotic disorders, including lung and liver fibrosis [18], supports the view that TGF-*β*1 pathway involvement in SG fibrogenesis may occur via promotion of the SG epithelial cell (SGEC) transition to form mesenchymal cells with a myofibroblast-like phenotype [19–21]. Immunohistochemical studies have demonstrated the expression of TGF-*β*1 by the glandular epithelium of healthy SGs, but in particular an increased expression in SGs of pSS patients [22]. However, the exact molecular mechanism that regulates the TGF-*β*1 pathway in pSS patients has not yet been elucidated. In this study, we investigated the relationship between TGF-*β*1/SMAD/Snail signaling and EMT-dependent fibrosis in pSS SGEC, using TGF-*β*1 treatment in human healthy SGEC as control, and analyzed the importance of TGF-*β*1/SMAD proteins and EMT-related molecular markers in clinical specimens from pSS patients. Results presented here show that the TGF-*β*1/SMAD/Snail signaling pathway regulates EMT-dependent fibrosis in pSS SGEC.

## 2. Materials and Methods

### 2.1. Reagents Used for Cell Treatment and Antibodies

Recombinant human TGF-*β*1 was obtained from R&D Systems (Minneapolis, MN). The following antibodies were used for the study: mouse anti-human TGF-*β*1 monoclonal antibody (mAb) (1 : 50, Santa Cruz Biotechnology, Santa Cruz, CA, USA), goat anti-human Smad2/3 polyclonal Ab (pAb) (1 : 100, R&D Systems, Minneapolis, MN, USA), goat anti-human p-SMAD2/3 (Ser 423/425) pAb (1 : 100, Santa Cruz Biotechnology), mouse anti-human SMAD4 mAb (1 : 50, Santa Cruz Biotechnology), rabbit anti-human Snail mAb (1 : 50, Cell Signaling Technology, Danvers, MA), mouse anti-human *β*-actin mAb clone AC-15 (1 : 100, Sigma-Aldrich, St. Louis, MO), mouse anti-human E-cadherin mAb (1 : 100, Dako, Santa Clara, CA, USA), mouse anti-human vimentin mAb (1 : 100, Thermo Fisher Scientific, Waltham, MA, USA), and rabbit anti-human collagen type I pAb (1 : 500, Thermo Fisher Scientific, Waltham, MA, USA).

### 2.2. Patient Selection and Characteristics

The Department of Pathology, University of Bari Medical School, selected 22 pSS labial SG (LSG) biopsies (patients aged 66.8 ± 2.3 years) according to the developed 2016 ACR/EULAR classification criteria for pSS [23]. These are based on the evaluation of five items: anti-SSA/Ro antibody positivity and focal lymphocytic sialadenitis with a focus score of ≥1 foci/4 mm^2^, each scoring 3, and an abnormal ocular staining score of ≥5 (or van Bijsterveld score of ≥4), a Schirmer's test result of ≥5 mm/5 minutes, and an unstimulated salivary flow rate of ≥0.1 ml/minute, each scoring 1. Healthy volunteers (aged 60.1 ± 1.4 years, *N* = 10) with no salivary condition were included in the study as controls. Informed consent was obtained from all the subjects, and the study was approved by the local ethics committee.

### 2.3. Histochemistry

Serial 3 *μ*m sections of healthy and pSS formalin-fixed, paraffin-embedded minor SG (MSG) tissues were used for immunohistochemical staining. While rehydrating the deparaffinized sections in graded alcohol, the slides were immersed for 1 hr in 70% ethanol supplemented with 0.25% NH_3_, and rehydration was resumed by immersion in 50% ethanol for 10 min. After deparaffinization and dehydration, the slides were washed in phosphate-buffered saline (PBS) (pH 7.6 3 × 10 min), then immersed in EDTA buffer (0.01 M, pH 8.0) for 20 min in a water bath at 98°C to unmask antigens. The sections were immunolabeled according to the following procedure: blockade of endogenous peroxidase by treatment with 3% hydrogen peroxide solution in water for 10 min at room temperature (RT); rinsing for 3 × 10 min in PBS, pH 7.6; preincubation in nonimmune donkey serum (Dako LSAB Kit, Dako, CA, USA) for 1 h at RT; and incubation overnight at 4°C with primary Abs at the dilutions indicated above. The sections were washed for 3 × 10 min in PBS and then incubated with the relative secondary Abs (Santa Cruz Biotechnology, TX, USA) diluted 1 : 200 in PBS for 1 h at RT, rinsed for 3 × 10 min in PBS, incubated with the streptavidin-peroxidase complex (Vector Laboratories, CA, USA) for 1 h at RT, incubated with the chromogen 3,3-diaminobenzidine tetrahydrochloride (DAB) (Vector Laboratories) for 10 min at RT, then counterstained with hematoxylin (Merck Eurolab, Dietikon, Switzerland). Negative controls of the immunoreactions were performed by replacing the primary Ab with donkey serum diluted 1 : 10 in PBS. After the addition of the secondary Ab, no specific immunostaining was observed in the negative controls (data not shown).

### 2.4. Histological Procedures for Collagen Expression Analysis

To detect collagen expression, serial 3 *μ*m sections of healthy and pSS formalin-fixed, paraffin-embedded MSG tissues were used and stained with Masson's trichrome stain for detection of collagen fibers.

### 2.5. Aperio Digital Immunohistochemistry Analysis and Quantification

Scans of stained tissue were obtained using a high-resolution digital Aperio Scanscope CS2 (Leica Biosystems, Nußloch, Germany), and an archive of the digital high resolution images was created. Digital slides were analyzed with Aperio ImageScope v.11 software (Leica Biosystems) at 10x magnification, and ten fields with an equal area were randomly selected for analysis at 40x magnification [24]. The expression of TGF-*β*1, p-SMAD2/3, SMAD4, Snail, E-cadherin, and vimentin was assessed with the Positive Pixel Count algorithm embedded in Aperio ImageScope software and reported as positivity percentage, defined as the number of positively stained pixels on the total pixels in the image. This approach allows a reliable automatic estimation of the amount of staining in the tissue, in addition to offering the benefit of reducing variability associated to human error [25].

### 2.6. Cell Culture and Treatment

Healthy subjects and pSS patients were subjected to the MSG explant outgrowth technique from the lower lip [26]. The tissues were dissociated by enzymatic and mechanical means into a suspension of single cells and plated onto a culture flask. pSS dispersal cells were resuspended in McCoy's 5a modified medium supplemented with 10% heat-inactivated (56°C for 30 min) FCS, 1% antibiotic solution, 2 mM L-glutamine, 50 ng ml^−1^ epidermal growth factor (EGF, Promega, Madison, WI, USA), and 0.5 *μ*g ml^−1^ insulin (Novo, Bagsværd, Denmark) and incubated at 37°C, 5% CO_2_ in air. The contaminating fibroblasts were selectively removed using 0.02% EDTA treatment. Immunocytochemical confirmation of the epithelial origin of cultured cells was routinely performed, as previously described [27]. Healthy human SGEC were grown in the same modified McCoy's 5A medium (Invitrogen) supplemented with 1% heat-inactivated FCS (to avoid excessive growth during treatment with TGF-*β*1). Healthy SGEC were stimulated with 10 ng/ml of TGF-*β*1 in the growth medium for 24–48 hours and then harvested for future analyses. To inhibit TGF-*β*1-dependent EMT, SB-431542 (R&D Systems, Minneapolis, MN, USA) was dissolved at a concentration of 10 mM in DMSO. TGF-*β*1-treated healthy human SGEC were incubated in medium supplemented with 10 mM of SB-431542 [28] at 37°C and 5% CO_2_ atmosphere. All experiments were performed in triplicate and repeated three times. Several dosage groups of SB431542 were designed to evaluate the inhibitory effect on TGF-*β*1.

### 2.7. Amplification of Gene Transcripts by Reverse Transcriptase Polymerase Chain Reaction (RT-PCR) and Quantitative Real-Time PCR (q-RT-PCR)

Total RNA from pSS SGEC, untreated healthy SGEC, TGF-*β*1-24 h-treated healthy SGEC, and TGF-*β*1 + SB-431542-24 h-treated healthy SGEC was isolated using the TRIzol reagent (Invitrogen Corp.). First-strand cDNA was synthesized by M-MLV reverse transcriptase (Promega, Madison, WI, USA) with 1 *μ*g each of DNA-free total RNA sample and oligo-(dT)15 (Life Technologies, Grand Island, NY, USA). Equal amounts of cDNA were subsequently amplified by PCR in a 20 *μ*l reaction mixture containing 2 *μ*M of each sense and antisense primer, PCR buffer, 2.4 mM MgCl_2_, 0.2 mM each dNTP, 10 *μ*l of transcribed cDNA, and 0.04 U/*μ*l Taq DNA polymerase. The primers used to amplify cDNA fragments were as follows: SMAD2, forward 5′-ACTAACTTCCCAGCAGGAAT-3′ and reverse 5′-GTTGGTCACTTGTTTCTCCA-3′; SMAD3, forward 5′-ACCAAGTGCATTACCATCC-3′ and reverse 5′-CAGTAGATAACGTGAGGGAGCCC-3′; SMAD4, forward 5′-TCCTGTGGCTTCCACAAGTC-3′ and reverse 5′-TCCAGGTGGTAGTGCTGTTATG-3′; Snail, forward 5′-ACCACTATGCCGCGCTCTT-3′ and reverse 5′-GGTCGTAGGGCTGCTGGAA-3′; E-cadherin, forward 5′-TTCCCTGCGTATACCCTGGT-3′ and reverse 5′-GCGAAGATACCGGGGGACACTCATGAG-3′; vimentin, forward 5′-AGGAAATGGCTCGTCACCTTCGTGAATA-3′ and reverse 5′-GGAGTGTCGGTTGTTAAGAACTAGAGCT-3′; and collagen type I, forward 5′-GAGCGGAGAGTACTGGATCG-3′, reverse 5′-TACTCGAACGGGAATCCATC-3′. The PCR cycling profile consisted of an initial denaturation step at 95°C for 15 min, followed by 35 cycles of 94°C for 60 s, 55°C for 60 s, and 72°C for 60 s. After PCR, amplification products were electrophoresed on 1.5% agarose gel containing ethidium bromide and visualized under ultraviolet transillumination. For qRT-PCR, forward and reverse primers for all the genes tested and the internal control gene *β*-2 microglobulin (part n° 4326319E; *β*2M) were purchased from Applied Biosystems (Assays-On-Demand, Applied Biosystems). Each qPCR reaction was run in triplicate on an ABI PRISM 7700 sequence detector (Applied Biosystems). Relative gene mRNA expression ratios were calculated using the ΔΔCt formula where Ct is the threshold cycle time value. The different expression of mRNA was deducted from 2^−ΔΔCt^.

### 2.8. Data Evaluation and Sequence Analysis

mRNA quantification data were based on the average of a set of three independent experiments, performed by gel image software (Bio-Profil Bio-1D; LTF Labortechnik GmbH, Wasserburg, Germany), and the intensity values for each band relative to GAPDH as reference were determined then expressed as arbitrary units. The identity of each PCR product was confirmed by the size, and the purified products were directly sequenced using the gene-specific forward or reverse primers.

### 2.9. Western Blot Analysis

Total cell lysates from pSS SGEC, untreated healthy SGEC, TGF-*β*1-24 h-treated healthy SGEC and TGF-*β*1 + SB-431542-24 h-treated healthy SGEC were prepared using a buffer (200 *μ*l) containing 1% Triton X-100, 50 mM Tris-HCl (pH 7.4), 1 mM PMSF, 10 *μ*g/ml soybean trypsin inhibitor, and 1 mg/ml leupeptin. The homogenates were centrifuged at 10,000 × *g* for 10 min at 4°C, and Bradford's protein assay was used to determine the protein concentrations. Equal amounts of samples were resolved by electrophoresis on 10% SDS-polyacrylamide gels, transferred to nitrocellulose membranes, and blotted under the following conditions: 200 mA (constant amperage) and 200 V for 110 min. Blots were blocked by phosphate-buffered saline (PBS) pH 7.2 with 0.1% (*v*/*v*) Tween 20 and 5% *w*/*v* nonfat dried milk for 1 h and washed three times with 0.1% (*v*/*v*) Tween 20-PBS 1x (T-PBS) and membranes were sequentially probed with the appropriate primary antibodies: goat anti-human Smad2/3 polyclonal Ab (pAb) (1 : 100, R&D Systems, Minneapolis, MN, USA), goat anti-human p-SMAD2/3 (Ser 423/425) pAb (1 : 100, Santa Cruz Biotechnology), mouse anti-human SMAD4 mAb (1 : 50, Santa Cruz Biotechnology), rabbit anti-human Snail mAb (1 : 50, Cell Signaling Technology, Danvers, MA), mouse anti-human E-cadherin mAb (1 : 100, Dako, Santa Clara, CA, USA), mouse anti human-vimentin mAb (1 : 100, Thermo Fisher Scientific, Waltham, MA, USA), and rabbit anti-human collagen type I pAb (1 : 500, Thermo Fisher Scientific, Waltham, MA, USA). The signals were developed with the chemoluminescence luminal reagent (Santa Cruz Biotechnology) according to the protocol. After incubation with a stripping buffer (Thermo Scientific, Middletown, VA, USA), immunoblots were probed with mouse anti-human *β*-actin mAb clone AC-15 (1 : 100, Sigma-Aldrich, St. Louis, MO; 0.25 *μ*g/ml) used as protein loading control.

### 2.10. Statistical Analysis

Data were subjected to statistical analyses by calculation of the mean percentage ± SE. Differences among groups were determined using *T*-test by Excel 2007 software (Microsoft). Statistical significance was set at ^∗^*p* < 0.05 and ^∗∗^*p* < 0.01. All experiments were performed a minimum of three times.

## 3. Results

### 3.1. Immunohistochemical Expression of the TGF-*β*1/SMAD Components in pSS Biopsies

**Figure 1:**
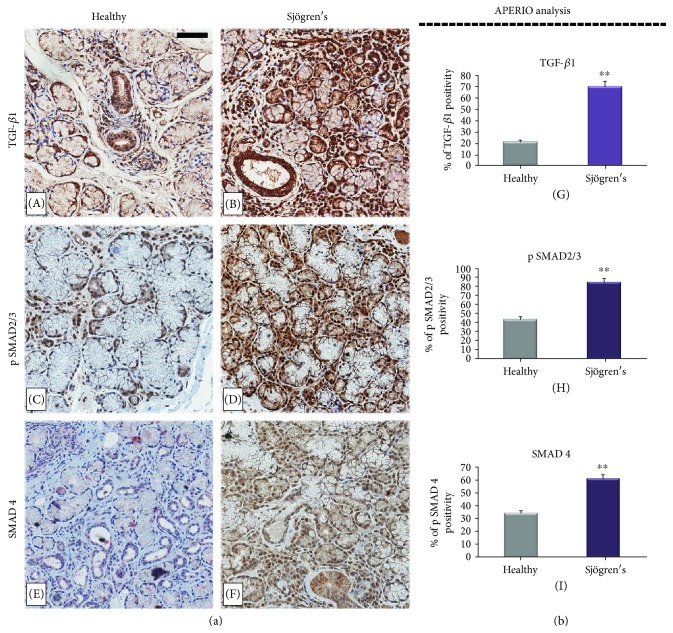
(a) Immunohistochemical localization of TGF-*β*1, pSMAD2/3, and SMAD4 in healthy (A, C, E) and pSS (B, D, F) SG tissues. The analysis revealed TGF-*β*1 expression in the cytoplasm of most interstitial infiltrating mononuclear cells, fibroblasts, and acinar and ductal cells of all the SG specimens analyzed. A strong positivity for TGF-*β*1 was detected in acinar and ductal cells of pSS sections (B), and the intensity of labeling was strongly reduced in healthy salivary tissue (A); high levels of pSMAD2/3 were detected in pSS specimens, and expression analysis showed that the number of positive cells for pSMAD2/3 was higher in pSS samples (D) when compared with normal tissue (C). A prominent immunostaining for SMAD4 was observed in acinar and ductal cells of pSS biopsies (F) compared with healthy subjects (E). Brown staining shows positive immunoreaction; blue staining shows nuclei. (A, B, C, D, E, F) original magnification, ×20; bar = 20 *μ*m. All images were scanned and analyzed with Aperio ImageScope instrument. (b) panels G, H, and I represent immunohistochemistry signal quantification of TGF-*β*1, pSMAD2/3, and SMAD4 positivity, respectively, performed by the Aperio ImageScope Software on healthy and pSS SG sections. Absorbance measurements performed by Aperio confirmed the microscopy observation and showed that staining for TGF-*β*1, pSMAD2/3, and SMAD4 were significantly increased in pSS glands than in the control glands (^∗∗^*p* < 0.01).

To determine the status of TGF-*β*1/SMAD signaling in human pSS SGs, we examined the expression of TGF-*β*1, pSMAD2/3, and SMAD4 by immunohistochemistry (IHC) in pSS biopsy samples, comparing them with healthy specimens (Figure 1).

TGF-*β*1 was detected in the cytoplasm of most interstitial infiltrating mononuclear cells, fibroblasts, endothelial cells, and duct cells in all the SG specimens analyzed. Acinar cell expression was weak except for a small number of acini in labial glands from patients with pSS. The cytoplasm of the few lymphocytes in normal glands expressed these growth factors, but in areas containing large periductal lymphocyte foci in primary pSS glands, and where there was confluent lymphocyte infiltration in SGs, staining was variable, some cells showing strong cytoplasmic expression and others being negative. In all specimens, TGF-*β*1 glandular expression was more pronounced in ductal epithelium than in acinar cells (panels A and B). In addition, clear differences in glandular expression of TGF-*β*1 between pSS and the corresponding SG control tissues appeared. Ductal epithelium in pSS glands appeared to stain darker than in normal control glands (Figure 1, A and B). Absorbance measurements performed by Aperio (Figure 1, B) confirmed this observation and showed that staining for TGF-*β*1 was significantly darker in pSS glands than in the control glands (69.9 ± 2.65 versus 20.8 ± 1.65, *p* < 0.01). Acinar staining was variable, staining being most pronounced in some acini adjacent to inflammatory cell foci.

To further investigate the integrity of SMAD signaling in pSS, we evaluated the percentage of pSMAD2/3-positive cells and the localization of this protein at the subcellular level in SG tissues derived from patients and healthy subjects (Figure 1). Immunohistochemical analysis of pSMAD2/3 expression showed that the percentage of positive cells for pSMAD2/3 was higher in pSS samples (84.6 ± 4.7) compared to normal tissue (43.4 ± 2.9) (*p* < 0.01) (Figure 1, C and D). Since SMAD4 interacts with pSMAD2/3 and participates in the intracellular TGF-*β*1 signaling pathway, its expression was immunohistochemically evaluated and a significantly increased expression for SMAD4 was noted in tubular and interstitial infiltrating mononuclear cells of pSS biopsies compared with healthy subjects (60.9 ± 2.78 versus 33.6 ± 3.87, *p* < 0.01). Figure 1 illustrates representative immunostaining for all markers and quantification by APERIO.

### 3.2. Expression of Snail and of the EMT-Related Molecular Markers in pSS Biopsies

**Figure 2:**
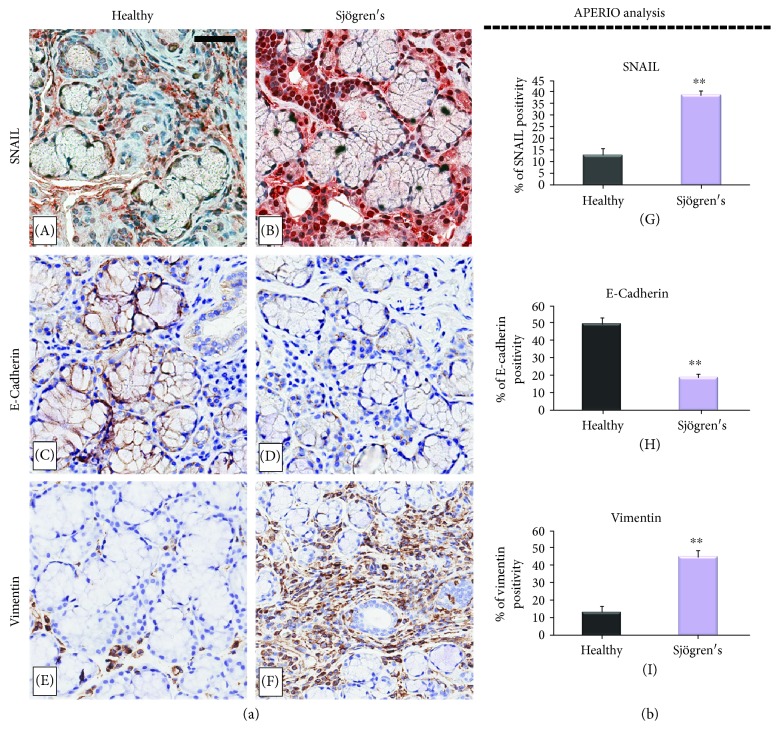
(a) EMT-related markers are significantly differentially expressed in pSS SGEC compared with healthy control tissues. The figure shows the immunohistochemical localization of SNAIL, E-cadherin, and vimentin in healthy (panels A, C, E) and pSS (panels B, D, F) SG biopsies. Clear differences in the immunostaining of Snail were detected in the nuclei of the acinar and ductal cells in pSS SG tissue (B) in comparison with healthy biopsies (A). E-cadherin was highly expressed in normal control SG tissues (C), and the expression of E-cadherin decreased strongly in diseased biopsies (D). A marked positivity for vimentin in pSS specimens was observed (F) than in healthy control SGs in which vimentin expression was significantly lower (E). Brown staining shows positive immunoreaction. (A, B, C, D, E, F) original magnification, ×20; bar = 20 *μ*m. All images were scanned analyzed with Aperio ImageScope instrument. (b) (G, H, I) represents immunohistochemistry signal quantification of Snail, E-cadherin, and vimentin, respectively, in healthy and pSS biopsies performed by the use of the computerized morphometric analysis software Aperio ScanScope and expressed in terms of pixel/intensities; graphs demonstrate a significant increased expression of Snail and vimentin and a reduction of E-cadherin in pSS (^∗∗^*p* < 0.01) (data represent mean ± SE of three independent experiments).

Snail, as a molecular organizer, resulted to be activated during the EMT pathway, determining the downregulation of the epithelial genes and upregulation of the mesenchymal genes. Therefore, to determine the mechanisms of TGF-*β*1-induced EMT in pSS SGEC, firstly the expression level of the Snail- and EMT-related molecular markers in the pSS SG tissue was analyzed, shown in Figure 2. As observed, a marked increase of Snail-labeled nuclei was observed in pSS specimens in comparison with healthy SGs (Figure 2, A and B). The brown signal was strongly expressed and confined to the nuclei of the acinar and ductal cells of the pSS tissues, and it was also detected in fibroblastic cells close to or surrounding the acini. By contrast, Snail staining detected in the nuclei of the acinar and ductal cells of the healthy salivary tissues was clearly weaker as compared with pSS tissues and poorly expressed in the fibroblasts.

During the EMT process, a Snail-dependent repression of E-cadherin and increased expression of vimentin also occur [29]. As expected, the expression changes of vimentin and E-cadherin revealed mesenchymal features. The results showed a strong positivity for Snail and vimentin in pSS specimens (Figure 2, B and F), and confirming the activation of EMT-related fibrosis, the expression levels of E-cadherin decreased in diseased SG biopsies (Figure 2).

**Figure 3:**
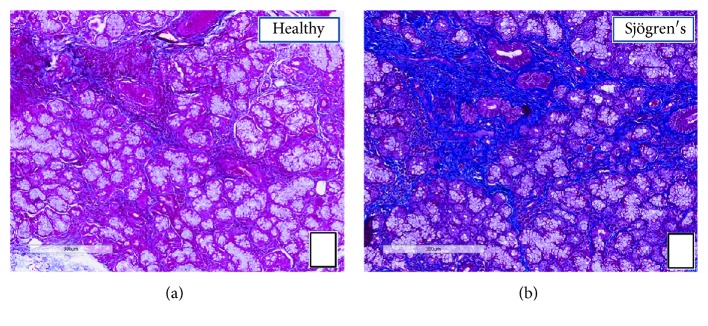
Histochemical analysis of collagen fiber deposition in SG tissues from healthy controls (a) and pSS (b) patients. The slides were subjected to Masson's trichrome staining to analyze collagen deposition (blue). At 10x magnification, a remarkable deposition of collagen fibers was seen in the interstitial area around acinar and ductal cells in pSS SG tissues (b), as compared to controls (a). Scale bar = 300 *μ*m.

Compared to the expression levels of molecular markers in the group of normal SG tissue, an upregulated expression of Snail and vimentin was observed in the group of pSS tissue, while the expression of E-cadherin was lower, as shown in Figure 2 (D). The expression of labeled Snail, vimentin, and E-cadherin proteins in the healthy and pSS biopsies obtained by the Aperio ScanScope was also quantified using computerized morphometric analysis software and expressed in terms of pixel/intensities (Figure 2(b), G, H, I), demonstrating a significantly (*p* < 0.01) increased expression of Snail and vimentin and a reduction of E-cadherin in pSS (Snail expression in pSS samples = 38.9 ± 1.7 versus normal tissue = 12.9 ± 2.9, *p* < 0.01; E-cadherin expression in pSS samples = 18.9 ± 1.8 versus normal tissue = 49.7 ± 3.3, *p* < 0.01; vimentin expression in pSS samples = 44.9 ± 3.8 versus normal tissue = 13.3 ± 3.2, *p* < 0.01). To assess collagen expression as index of increased fibrosis in pSS, Masson's trichrome staining was used (Figure 3, A and B). Fibrotic areas are rich in collagen s, and therefore, they appear in blue upon Masson trichrome staining. We observed that the total collagen density was lower in healthy normal SG tissue (panel A) than in pSS specimens (panel B). These results are consistent with previous results indicating that EMT-dependent fibrosis occurs in pSS.

### 3.3. TGF-*β*1 Induces EMT-Related Changes in SGEC

**Figure 4:**
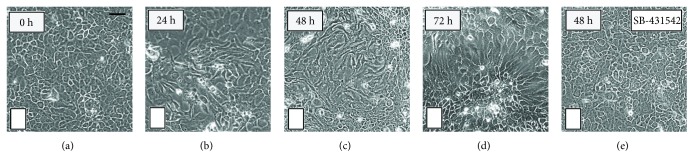
Morphological changes in healthy SGEC induced by TGF-*β*1 treatment. SGEC derived from SG biopsies of healthy donors were incubated with 10 ng/ml of TGF-*β*1 for 24–72 h. (a) Untreated SGEC show a clear pebble-like shape and cell-cell adhesion. TGF-*β*1-treated cells (b, c, d) show a decrease in cell-cell contacts and adopt a more elongated morphological shape. Healthy SGEC stimulation with TGF-*β*1 (10 ng/ml) in the presence of SB-4315425 (10 *μ*M) for 48 h is reported in the panel (e). Changes in cell morphology were assessed under phase-contrast light microscopy (original magnification, ×20). Bar = 20 *μ*m.

To confirm the activation of TGF-*β*1-dependent EMT in pSS, *in vitro* analysis of the morphological changes indicative of the EMT was performed in SGEC exposed to TGF-*β*1, after different time intervals of incubation. TGF-*β*1 stimulation induced changes in cellular morphology; confluent SGEC in culture have, in fact, a typical epithelial morphology. However, after 72 hours of TGF-*β*1 exposure, the cells expressed a morphological phenotype characteristic of the EMT, featuring a loss of cell-cell contact, an elongated shape (Figure 4, B, C, D), and cytoskeletal alterations with an evident development of thick actin fibers (Figure 4). As observed, SB-431542 treatment attenuated the effect of TGF-*β*1 without obvious morphologic alterations on SGEC (Figure 4, E).

### 3.4. Alteration of Gene Expression Patterns Downstream of TGF-*β*1 in pSS

**Figure 5:**
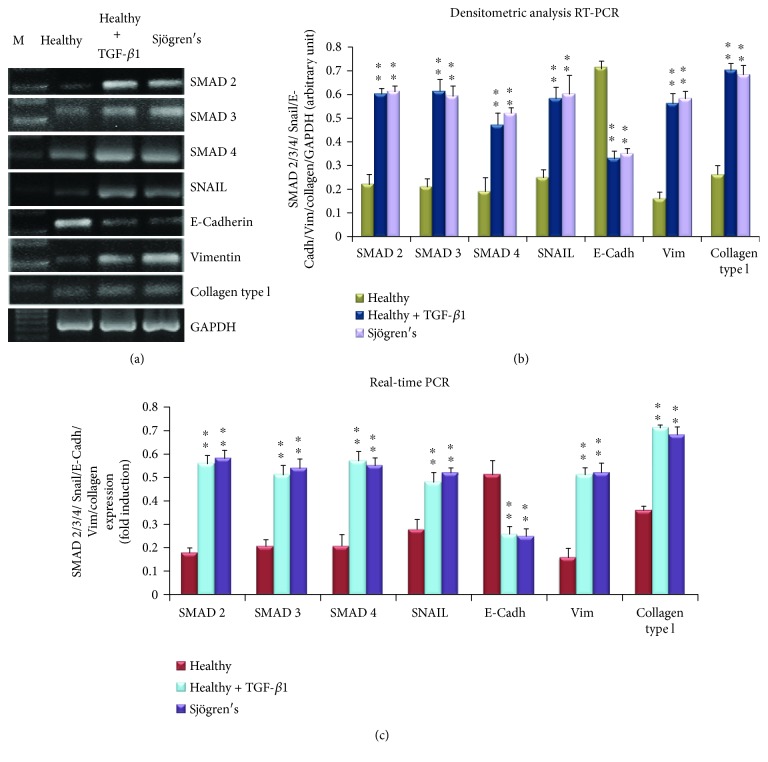
TGF-*β*1 induces transcription of the SMAD complex and EMT-related molecular markers in healthy SGEC. SMAD 2, 3, and 4, SNAIL, E-cadherin, vimentin, and collagen type I gene expression were quantified by semiquantitative RT-PCR (a, b) and real-time PCR (c) in healthy SGEC treated with TGF-*β*1 for 24 h, untreated control cells, and pSS SGEC. The images show that TGF-*β*1 treatment determined increased levels of SMAD 2, 3, and 4 and Snail gene expression comparable to those observed in pSS SGEC. The increasing gene expression of mesenchymal marker vimentin and collagen type I and the decreased gene expression of epithelial marker E-cadherin were also revealed. Band intensities were analyzed by densitometric analysis performed by gel image software, normalized against that of GAPDH, and expressed in arbitrary units (b). In the real-time PCR (c), *β*2-Microglobulin-normalized relative expression levels of SMAD 2, 3, and 4, SNAIL, E-cadherin, vimentin, and collagen type I mRNA are shown. Gene expression changes observed in real-time PCR analysis are in agreement with the results monitored by semiquantitative RT-PCR; SMAD 2, 3, and 4, Snail, vimentin, and collagen type I gene expression was increased upon treatment of healthy SGEC with TGF-*β*1, while E-cadherin resulted decreased (data represent mean ± SE; *n* = 3). Asterisks indicate statistical significance, *p* < 0.01. M = marker; Vim = vimentin; E-cadh = E-cadherin.

To demonstrate the activation of an EMT-dependent fibrosis program in TGF-*β*1-treated human SGEC, and in an attempt to determine whether any of the EMT genes showed pSS disease-specific patterns of expression, we compared the relative gene expression levels of *SMAD2/3/4*, Snail, E-cadherin, vimentin, and collagen type I using RNAs isolated from TGF-*β*1-24 hour-treated SGEC and pSS SGEC (Figure 5). In all samples examined, we found that the gene expression levels of *SMAD 2*, *3*, and *4* and Snail were up to double those observed in untreated SGEC; the same gene expression alteration was detected in pSS SGEC (Figure 5, A–C). Furthermore, as shown in Figure 5, TGF-*β*1 decreased E-cadherin mRNA expression from baseline values by over 30% at 24 h (*p* < 0.01) and increased vimentin and collagen type I gene expression (*p* < 0.01). Consistent with the gene expression data obtained upon TGF-*β*1 stimulation, in pSS SGEC we observed altered *SMAD2*, *3*, and *4*, *Snail*, *E-cadherin*, *vimentin*, and collagen type I gene expression in comparison with healthy SGEC, demonstrating biochemical changes consistent with an EMT-dependent pathway activation in diseased SGs (Figure 5).

To further confirm the differences in the TGF-*β*1 signaling pathway between TGF-*β*1-treated healthy SGEC, pSS SGEC, and untreated healthy SGEC, we analyzed the expression of *SMAD2*, *3*, and *4*, *Snail*, *E-cadherin*, *vimentin*, and collagen type I by real-time PCR (Figure 5(c)). This confirmed that all of these signaling genes are expressed in TGF-*β*1-treated SGEC and pSS SGEC, and these cells displayed higher mRNA expression than normal SGEC did, except for E-cadherin, whose mRNA expression was decreased in TGF-*β*1-treated SGEC and pSS SGEC as compared with healthy SGEC (*p* < 0.01) (Figure 5(c)). We concluded from these data that TGF-*β*1 induces the EMT in healthy SGEC, as revealed by a downregulation of epithelial markers and an upregulation of mesenchymal markers at the RNA level, and these results show that pSS SGEC can undergo EMT-dependent fibrosis in response, via a primarily SMAD-dependent mechanism.

### 3.5. TGF-*β*1-Induced EMT Activation Is Independent of the Canonical TGF-*β*/SMAD Pathway in pSS

**Figure 6:**
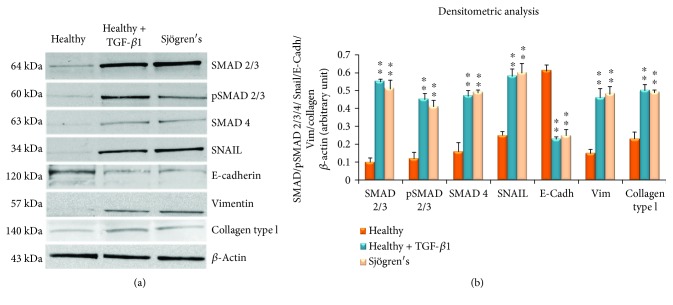
(a) Western blot analysis of TGF-*β*1/SMAD/Snail and of the mesenchymal markers E-cadherin, vimentin, and collagen type I in cultured untreated SGCE, TGF-*β*1- (10 ng/ml-) treated healthy SGEC for 48 hours, and pSS SGEC. Lane 1: healthy control SGEC, lane 2: TGF-*β*1-treated healthy SGEC, and lane 3: pSS SGEC. Protein expressions were quantified using ImageJ software (b). The data are expressed as relative intensity of proteins normalized to *β*-actin expression. Values are considered statistically significant at ^∗^*p* < 0.05 or ^∗∗^*p* < 0.01. In concordance with the RT-PCR and real-time PCR data, in TGF-*β*1-treated healthy SGEC, a significant increase of total SMAD 2/3, pSMAD 2/3, SMAD 4, SNAIL, vimentin, and collagen type I band intensity was observed in comparison with untreated control healthy SGEC, while epithelial marker E-cadherin band intensity was reduced compared with untreated control SGEC. In addition, Western blot images related to pSS SGEC completely overlapped with Western blot results obtained from healthy SGEC stimulated with the TGF-*β*1. Immunoblotting gave rise to bands of the expected size. All Western blots were repeated a minimum of three times.

The results of gene expression were in accordance with Western blotting findings (Figures 6(a) and 6(b)): SMAD 2/3, p-SMAD2/3, SMAD 4, and Snail proteins showed very limited expression in the untreated SGEC control group and increased at 48 h after TGF-*β*1 stimulation of the SGEC. Western blotting indicated, furthermore, that treatment of SGEC cells with 5 ng/ml TGF-*β*1 for 48 h led to a significant reduction in the epithelial phenotype marker E-cadherin and an increase in the mesenchymal phenotype markers vimentin and collagen type I (Figures 6(a) and 6(b)). All these results indicate that the EMT can be induced by TGF-*β*1 in healthy SGEC. Notably, the Western blot images related to the TGF-*β*1-stimulated SGEC correlated very well with Western blot results obtained for pSS SGEC (Figures 6(a) and 6(b)), suggesting that a similar mechanism could be proposed for the activation of the TGF-*β*1-dependent EMT observed in pSS SGEC.

**Figure 7:**
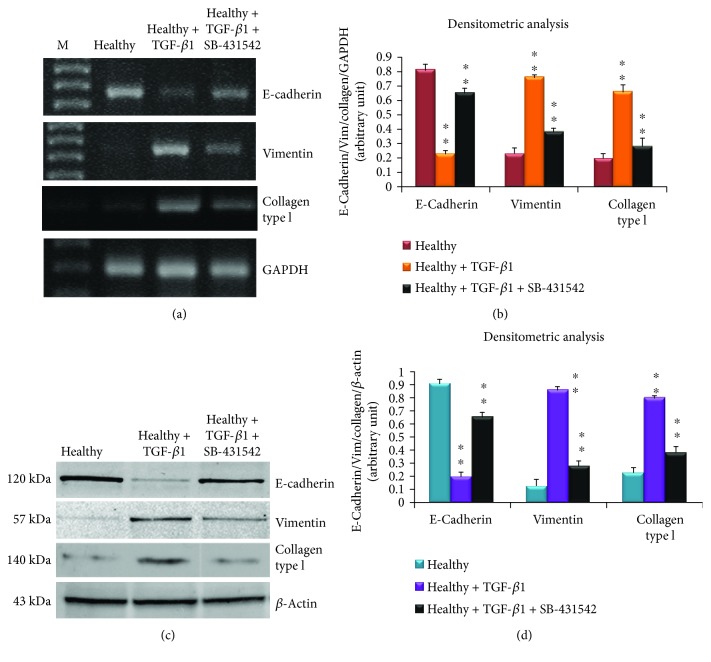
Effects of SB-431542 treatment on the expression of E-cadherin, vimentin, and collagen type by RT-PCR and Western blot. (a, b) E-cadherin, vimentin, and collagen type I gene expression was quantified by semiquantitative RT-PCR in healthy SGEC treated with TGF-*β*1 + SB-431542 (10 *μ*M) or TGF-*β*1 (10 ng/ml) alone as control. Band intensities were analyzed by densitometric analysis performed by gel image software, normalized against GAPDH, and expressed in arbitrary units (b). (c, d) Effects of SB-431542 (10 *μ*M) addition on the expression of E-cadherin, vimentin, and collagen type I in TGF-*β*1-treated healthy SGEC by Western blot. *Y* axis of (d) represents the banding densities of samples tested versus *β*-actin. Data are expressed as a significant change relative to TGF-*β*1-treated healthy SGEC. Each bar represents mean ± SE (^∗^*p* < 0.05 and ^∗∗^*p* < 0.01); each experiment was repeated three times.

To further confirm the essential role of TGF-*β*1 in the EMT process activation, we treated healthy SGEC with TGF-*β*1 + SB-431542. After SB-431542 treatment, E-cadherin gene synthesis increased, while vimentin and collagen type I gene expression decreased (Figures 7(a) and 7(b), *p* < 0.01). Similarly, E-cadherin, vimentin, and collagen type I protein expression in response to SB-431542 treatment was evaluated by Western blot. Results obtained demonstrated that the E-cadherin, vimentin, and collagen type I expressions were significantly different in the SB-431542-treated SGEC cells as compared with healthy SGEC treated with TGF-*β*1 alone (Figures 7(c) and 7(d), *p* < 0.01, optical densities of test bands versus *β*-actin bands). Thus, SB-431542, as a specific inhibitor of the TGF-*β*1/Smad signaling pathway [28], attenuates TGF-*β*1-mediated EMT in human SGEC, confirming that the TGF-*β*1/SMAD signaling pathway regulates EMT in human SGEC.

## 4. Discussion

This study was the first to be conducted to confirm that TGF-*β*1 can induce the EMT in SGEC derived from pSS patients. We demonstrated that an aberrant upregulation of TGF-*β*1 in the pSS SGs causes morphological and functional mesenchymal changes in SGEC via the activation of the TGF-*β*1/SMAD/Snail signaling pathway, thereby contributing to the fibrotic process in the SGs. Furthermore, exogenous TGF-*β*1 in healthy cultured SGEC also induced pathological fibrotic sequelae in SGs, leading to evident morphological changes of the glandular epithelium.

Fibrosis is the final, common pathological outcome of many chronic inflammatory diseases. TGF-*β*1 is the primary factor that drives tissue fibrosis in most forms of these inflammatory conditions in different organs such as the liver [30], kidney [31–33], and lung [34]. TGF-*β*1 is able to encourage the EMT program in some cells undergoing a phenotypic transition to mesenchymal cells, often forming fibroblast-like cells that produce extracellular matrix components, thus participating in the fibrotic tissue formation [6, 35, 36]. Interestingly, excessive TGF-*β*1 production or boosting of its profibrotic effects induces marked fibrotic changes and contributes to a pathologic excess of tissue fibrosis, compromising normal organ function [37–40].

This study offers the first demonstration that the level of TGF-*β*1 is increased in SG tissues derived from biopsies of patients affected by pSS, characterized by a severe inflammatory condition that contributes to an excess of fibrotic tissue, compromising the functional morphology of SGs. Indeed, inflammation is recognized as the earliest stage of fibrosis, and long-term inflammation is associated with the development of fibrogenesis [41, 42]. In other words, inflammation is key to the onset of fibrosis.

Accumulating evidence suggests that TGF-*β*1 induces the EMT and promotes fibrosis in many kinds of tissues [37–40]. There is little current evidence that the EMT occurs in SGEC and that it plays a role in the SG fibrosis that accompanies the pSS. To address these uncertainties, we used several approaches to explore salivary EMT and to investigate the molecular mechanism triggered during the pathogenesis of salivary fibrosis.

The TGF-*β*1/SMAD/Snail pathway is a particularly interesting system implicated during the fibrotic process in a number of disease states [30–34, 43–47]. It is well-known that the TGF-*β*1 signal exerts its biological effects via type I and type II serine/threonine kinase receptors and the main signaling pathway is primarily mediated by phosphorylation of regulatory SMAD2/3, which forms a complex with the common coregulatory SMAD4. This SMAD complex then shuttles to the nucleus where, in cooperation with other transcription factors [48], it stimulates the transcription of target genes like the zinc finger transcription factor Snail; Snail plays an active role in guiding the EMT process by downregulating epithelial marker proteins such as E-cadherin and upregulating mesenchymal markers such as vimentin and collagen type 1 [48–52]. Peinado et al. demonstrated the ability of TGF-*β*1 to induce the EMT in Madin-Darby canine kidney cells and showed that Snail overexpression leads to a total repression of E-cadherin and the induction of a complete EMT, suggesting that Snail is a direct target of TGF-*β*1 signaling [53].

Supported by all these observations, we tested whether the TGF-*β*1 signaling pathway is triggered in human pSS SGs, firstly by immunohistochemistry. As expected, this analysis demonstrated that TGF-*β*1, pSMAD2/3, and SMAD4 proteins are widely expressed in the pSS tissue patients at higher expression levels compared with healthy SG tissues. Furthermore, our findings revealed a strong positivity for Snail, vimentin, and collagen type I in pSS specimens in comparison with normal SG tissue, while the expression levels of E-cadherin were decreased in diseased SG biopsies, evidencing that TGF-*β*1 induces the EMT in SGEC via the canonical TGF-*β*1/SMAD/Snail pathway.

Furthermore, phenotypic changes characteristic of the EMT in response to TGF-*β*1 treatment were observed in various cells, such as alveolar pulmonary epithelial cells [45]. Several in vitro studies have demonstrated that the addition of TGF-*β*1 to cultured human epithelial cells induces a downregulated E-cadherin expression and transformation to mesenchymal cells, resembling myofibroblasts [36].

To test this possibility, SGEC derived from healthy salivary biopsies of healthy donor volunteers were exposed to TGF-*β*1 treatment. The treated cells adopted a more fibroblast-like morphology and reduced their cell-cell contact, whereas when cultured in the absence of TGF-*β*1 or in the presence of the TGF-*β*1-pathway inhibitor SB-431542, they maintained a classic cobblestone epithelial morphology and growth pattern. Interestingly, TGF-*β*1 was able to induce the EMT in SGEC, probably by disrupting the integrity of cell-cell contacts, likewise indicating an involvement of E-cadherin in TGF-*β*1-mediated EMT. In this context, we compared the relative gene and protein levels of *SMAD2/3/4*, Snail, E-cadherin, vimentin, and collagen type I using mRNAs and proteins isolated from TGF-*β*1-treated SGEC and pSS SGEC. Consistent with the above research, we found, both at gene and protein level, higher expression levels of *SMAD2*, *3*, and *4* and Snail in the SGEC treated with TGF-*β*1 compared to untreated healthy SGEC.

**Figure 8:**
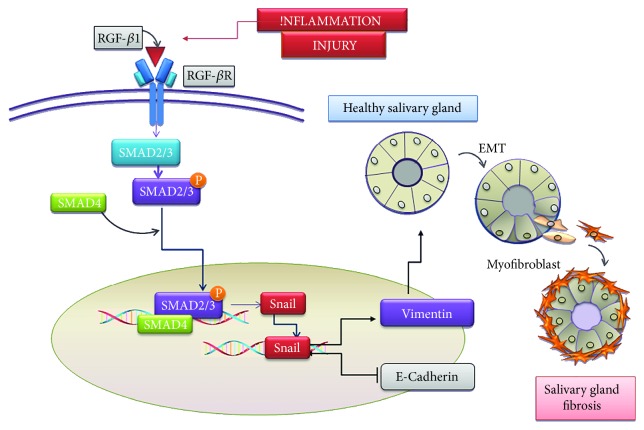
Schematic illustration of a working model for the TGF-*β*1/SMAD/Snail pathway in modulation of EMT-dependent fibrosis in SGEC. TGF-*β*1 stimulates epithelial cells by binding and activating transmembrane kinase receptors leading to phosphorylation and activation of Smad2/3. Once activated, pSmad2/3 form heterocomplexes with Smad4, which collectively translocate to the nucleus to mediate signaling events linked to EMT activation. The activation of the transcription factor Snail and induction of EMT markers have, as consequence, the upregulation of the mesenchymal marker Vimentin and the downregulation of the epithelial marker E-cadherin. During the activated EMT program, SGEC exhibit dramatic morphological changes and the gain of mesenchymal properties including increased migratory capacity and contractility. Finally, these mesenchymal cells become myofibroblasts which are responsible for progressive SG fibrosis.

Furthermore, in the TGF-*β*1-treated samples examined, we found a significant reduction in the epithelial phenotype marker E-cadherin and an increase in the mesenchymal phenotype markers, vimentin and collagen type I, as compared to those observed in untreated SGEC, indicating that TGF-*β*1 induces the EMT via the TGF-*β*1/SMAD signaling pathway. Based on our findings, a possible scheme for the TGF-*β*1/SMAD/Snail signaling pathway in pSS SGEC is shown in Figure 8.

## 5. Conclusions

We conclude that the high level of TGF-*β*1 in pSS salivary gland tissues suggests that TGF-*β*1 is an important factor in the transition phase from SG inflammation to SG fibrosis, characterized by changes in the morphology and function of TGF-*β*1-treated healthy SGEC. This was confirmed by the altered expression of epithelial markers and mesenchymal markers at the gene and protein levels, demonstrating that pSS SGEC can undergo EMT-dependent fibrosis in response to a TGF-*β*1/SMAD/Snail-dependent mechanism. Further study of the function of TGF-*β*1 in the fibrotic process of the SGs is warranted to develop new therapies to arrest the development of salivary fibrosis at an early stage.

## Data Availability

The data used to support the findings of this study are included within the article.
